# Integrated Single-Cell and Transcriptome Sequencing Analyses Identify Dipeptidase 2 as an Immune-Associated Prognostic Biomarker for Lung Adenocarcinoma

**DOI:** 10.3390/ph16060871

**Published:** 2023-06-12

**Authors:** Xiangqian Zhang, Yunfan Zhang, Xiaomei Gao, Ye Zhang, Yongheng Chen

**Affiliations:** 1 NHC Key Laboratory of Cancer Proteomics & State Local Joint Engineering Laboratory for Anticancer Drugs, Department of Oncology, National Clinical Research Center for Geriatric Disorders, Xiangya Hospital, Central South University, Changsha 410008, China; 2 College of Life Sciences, Hunan Normal University, Changsha 410008, China; 3 Department of Pathology, Xiangya Hospital, Central South University, Changsha 410008, China

**Keywords:** lung adenocarcinoma, immune infiltration, dipeptidase 2, macrophage, immunotherapy

## Abstract

Dipeptidase 2 (DPEP2) is a dipeptidyl peptidase that plays an important role in the hydrolysis of leukotriene D4 (LTD4) to leukotriene E4 (LTE4). Previous studies have suggested that LTD4 promotes tumor progression and survival in non-small cell lung cancer (NSCLC). Therefore, we hypothesized that DPEP2 may play a pivotal role in this tumor. Given that lung adenocarcinoma (LUAD) is the most common subtype of NSCLC, our study aimed to examine the expression and function of DPEP2 in LUAD. Based on bioinformatics and the analysis of clinical samples, our findings revealed that DPEP2 is highly expressed in normal lung tissues, but downregulated in LUAD tissues, and its expression levels were significantly associated with clinical indicators of tumor grade and prognosis. Pathway enrichment analysis showed that DPEP2 is involved in biological processes such as chemokine signaling pathways, leukocyte trans-endothelial migration, and humoral immune responses in LUAD. In addition, DPEP2 expression was significantly associated with various immune cells, especially monocytes–macrophages. Single-cell transcriptome data further confirmed the expression of DPEP2 dominantly in macrophages from normal lung tissues. Analysis of the TCIA database revealed that high DPEP2 expression is associated with a stronger response to immune checkpoint inhibitors such as CTLA4 and PD1, and determines sensitivity to LUAD therapeutic agents. Furthermore, we found that DPEP2 inhibits the migration and invasion of LUAD cells. Therefore, DPEP2 may serve as a potential immune biomarker and therapeutic target for LUAD, providing novel therapeutic approaches for this disease.

## 1. Introduction

Lung cancer remains the leading cause of global mortality, highlighting its significant impact on public health [1]. Lung adenocarcinoma (LUAD) is the predominant histological subtype of non-small cell lung cancer (NSCLC), representing 80% of all lung cancer cases globally [2]. Despite significant progress in the diagnosis and treatment of LUAD in recent decades, the prognosis for patients with LUAD remains bleak [3]. Research on genes, miRNAs, lncRNAs, and other molecular factors is crucial for advancing early diagnosis and personalized treatment approaches [4]. Thus, there is a pressing need to identify novel biomarkers and therapeutic targets.

Leukotriene D4 (LTD4) is a bioactive lipid molecule with multifaceted roles in inflammation [5], immunity [6], and various cancers [7,8], including LUAD [9]. By binding to its receptors, CysLT1R and CysLT2R, LTD4 modulates tumor cell growth, proliferation, invasion, and metastasis [10]. Recent studies have highlighted the pivotal role of the LTD4/CysLT1R signaling pathway in driving LUAD progression [9]. Nonetheless, the underlying mechanism through which LTD4 exerts its effects in LUAD remains unclear.

Dipeptidase 2 (DPEP2), a cell membrane enzyme that metabolizes LTD4 to leukotriene E4 (LTE4) [11], may offer a promising therapeutic target for modulating the LTD4-mediated signaling pathway in LUAD. Previous reports suggest that DPEP2 acts as an inflammatory regulator of macrophages and may have a protective effect against CVB3-induced viral myocarditis [12]. DPEP2 has also been identified as a potential biomarker for diabetic retinopathy [13]. In respiratory diseases, DPEP2 has been implicated in chronic rhinitis, sinusitis, and asthma [14]. However, the role of DPEP2 in respiratory tumors, particularly in LUAD, has yet to be fully elucidated. Interestingly, bioinformatics analyses have suggested that DPEP2 is a valuable independent prognostic marker for predicting patient survival and prognosis in LUAD. Furthermore, downregulation of DPEP2 expression has been observed in LUAD, hinting at its potential involvement in the pathogenesis and progression of this malignancy. Thus, elucidating DPEP2’s biological functions and potential mechanisms in LUAD is a critical area of research with significant implications for diagnosis and treatment.

## 2. Results

### 2.1. DPEP2 Expression Is Down-Regulated in LUAD

**Figure 1:**
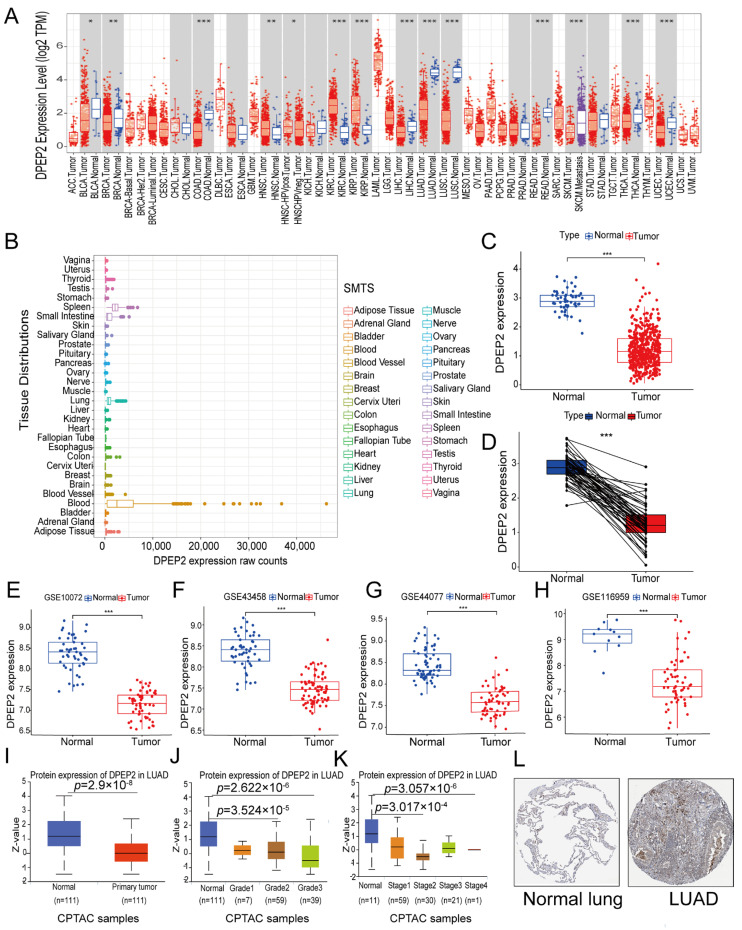
The expression level of DPEP2 is downregulated in LUAD. (**A**) The mRNA expression levels of DPEP2 in pan-cancer from the TIMER database. (**B**) Expression levels of DPEP2 in multiple normal tissue types according to the GTEX database. (**C**) Differential expression of DPEP2 in normal and tumor TCGA-LUAD samples. (**D**) DPEP2 pairwise difference analysis in normal and tumor TCGA-LUAD samples. (**E**–**H**) Validation of normal DPEP2 tissues versus tumor tissues across four GSE datasets. (**I**–**K**) Using the UALCAN database, changes in DPEP2 protein levels between normal lung tissue and LUAD tissue were explored, as well as changes in grades and stages. (**L**) The THPA database validates DPEP2 expression levels in LUAD and non-tumor tissues. (*p < 0.05, ** *p* < 0.01, *** *p* < 0.001).

We retrieved data from the TIMER database to investigate the expression of DPEP2 in pan-cancer. Our analysis revealed that DPEP2 expression was significantly lower in many malignant tumors, particularly in LUAD and lung squamous cell carcinoma (LUSC), than in normal tissues (Figure 1A). Furthermore, analysis of the GTEx database demonstrated that DPEP2 was highly expressed in normal lung tissue relative to heart and kidney tissues and exhibited relatively elevated levels in the spleen and small intestine (Figure 1B). In this study, LUAD was of particular interest to us. Therefore, we further assessed the expression levels of DPEP2 in LUAD using TCGA and GEO data. Our results demonstrated a significant decrease in DPEP2 mRNA expression in LUAD tissues relative to normal lung tissue (Figure 1C–H). In addition, we investigated the protein expression levels of DPEP2 in LUAD tissues and normal lung tissues, as well as the differences in protein levels between different grades and stages of LUAD, and these differences were significant (Figure 1I–K). We finally confirmed the downregulation of DPEP2 in LUAD tissues by analyzing immunohistochemical staining images of LUAD obtained from the THPA database (Figure 1L). Overall, these results suggest that the expression of DPEP2 is higher in normal tissues compared to LUAD tissues. (*p < 0.05, ** *p* < 0.01, *** *p* < 0.001).

### 2.2. Relationships between DPEP2 and Clinicopathological Characteristics of LUAD Patients

Based on the median DPEP2 mRNA levels, the TCGA-LUAD cohort was divided into two groups: 268 patients in the high-DPEP2 expression group and 267 patients in the low-DPEP2 expression group. We compared the differences in clinical characteristics between the two groups. Table 1 shows that in LUAD patients, DPEP2 expression was significantly correlated with T stage, N stage, pathologic stage, age, smoker, and OS event (all *p* < 0.05). However, there was no statistically significant relationship with other clinicopathological factors, such as M stage (*p* value = 0.552).

### 2.3. DPEP2 Is a Favorable Independent Prognostic Factor for LUAD

**Figure 2:**
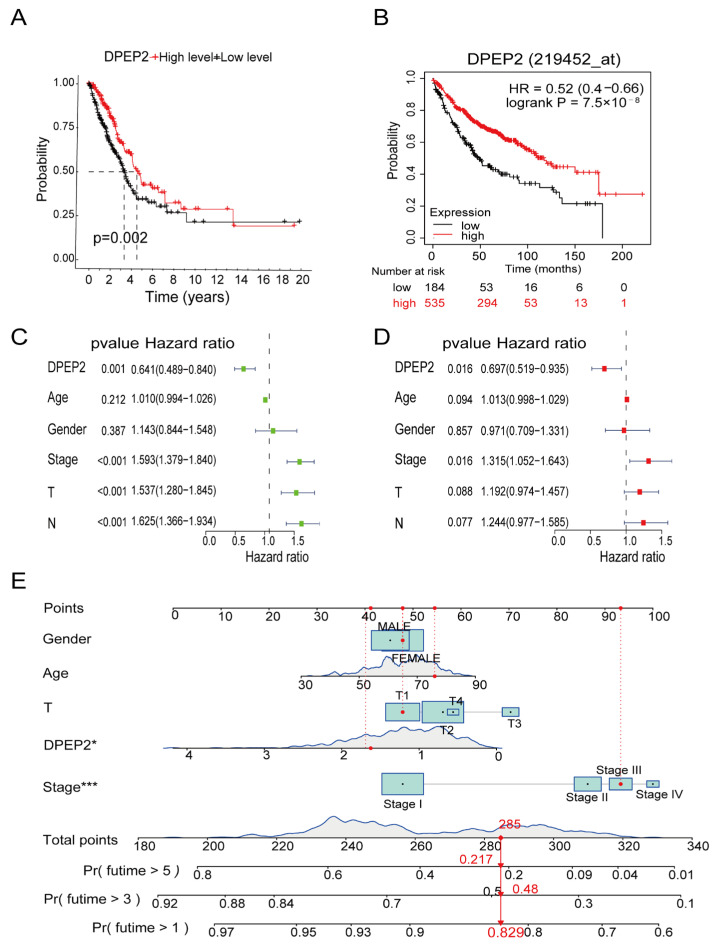
High expression of DPEP2 is associated with better survival in LUAD patients. (**A**) Survival analysis of LUAD patients in the DPEP2 high expression group and the DPEP2 low expression group using survival data from TCGA-LUAD. (**B**) Survival analysis of LUAD patients in the DPEP2 high expression group and the DPEP2 low expression group using the K–M plotter database. (**C**,**D**) Univariate Cox analysis and multivariate Cox analysis in the TCGA-LUAD cohort, with stage and DPEP2 expression as independent prognostic factors. (**E**) Establishing nomograms based on DPEP2 expression and clinical characteristics to predict the survival probability of LUAD patients at 1, 3, and 5 years. (*p < 0.05, ** *p* < 0.01, *** *p* < 0.001).

The results of survival analysis showed that the high DPEP2 expression group favored the survival of TCGA-LUAD patients (*p* value = 0.002) (Figure 2A), and the K–M plotter database showed the same results (HR = 0.54, log-rank *p* =7.5 × 10^−8^) (Figure 2B). Furthermore, univariate analysis revealed that stage, T stage, N stage, and DPEP2 expression were significantly correlated with the prognosis of patients with LUAD (Figure 2C). Additionally, multifactorial analysis demonstrated that stage and DPEP2 expression were significantly associated with the prognosis of patients with LUAD (Figure 2D). The above results suggest that DPEP2 could be a favorable independent prognostic factor for LUAD. Moreover, based on gender, age, T stage, DPEP2 expression, and stage, a nomogram was developed to predict the survival of LUAD patients. The nomogram predicted survival rates for LUAD patients at one, three, and five years (Figure 2E). Calibration curves were used to evaluate the nomogram’s predictive efficacy (Figure S1).

### 2.4. GO, KEGG, and GSEA Identify DPEP2-Related Signaling Pathways in LUAD

**Figure 3:**
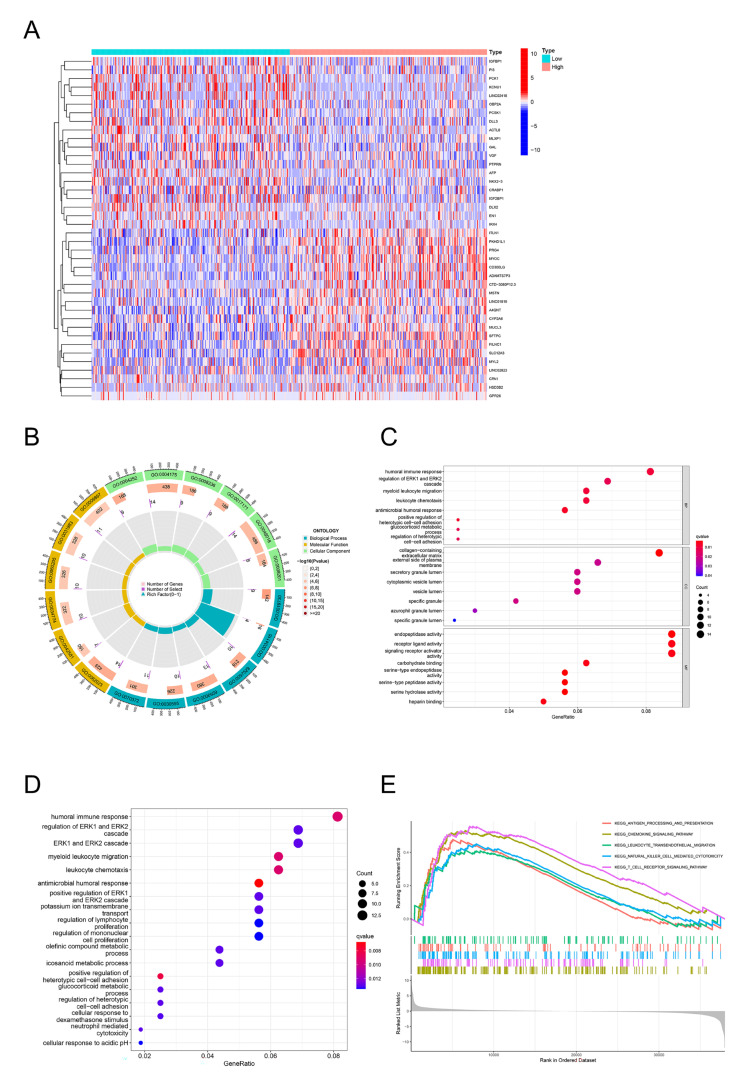
DPEP2-related genes are significantly enriched in multiple immune-related pathways. (**A**) The heatmap depicts the top 20 most significantly up- and downregulated genes in the high and low DPEP2 expression groups. (**B**,**C**) GO enrichment analysis. (**D**) KEGG enrichment analysis. (**E**) GSEA enrichment analysis.

In our study, we identified the 160 most significantly upregulated genes and 40 most significantly downregulated genes based on the analysis of variance thresholds. Table S1 contains detailed information about the DEGs. The heatmap shows the DEGs in the high and low DPEP2 expression groups (Figure 3A). We then performed GO, KEGG, and GSEA analyses using the significantly upregulated genes. GO analysis revealed that DPEP2 was mainly involved in biological processes such as antimicrobial humoral response, bone marrow leukocyte migration, humoral immune response, and leukocyte chemotaxis (Figure 3B,C). KEGG pathway analysis revealed myeloid leukocyte migration, positive regulation of heterotypic cell–cell adhesion, humoral immune response, leukocyte chemotaxis, etc. (Figure 3D). Antigen processing and presentation, the chemokine signaling pathway, leukocyte trans endothelial migration, natural killer cell-mediated cytotoxicity, and the T-cell receptor signaling pathway were all enriched in GSEA (Figure 3E). These findings suggest that DPEP2 has a broad impact on the prognosis and immune regulation of LUAD.

### 2.5. DPEP2 and Its Co-Expressed Genes Are Significantly Associated with Immunity in LUAD

**Figure 4:**
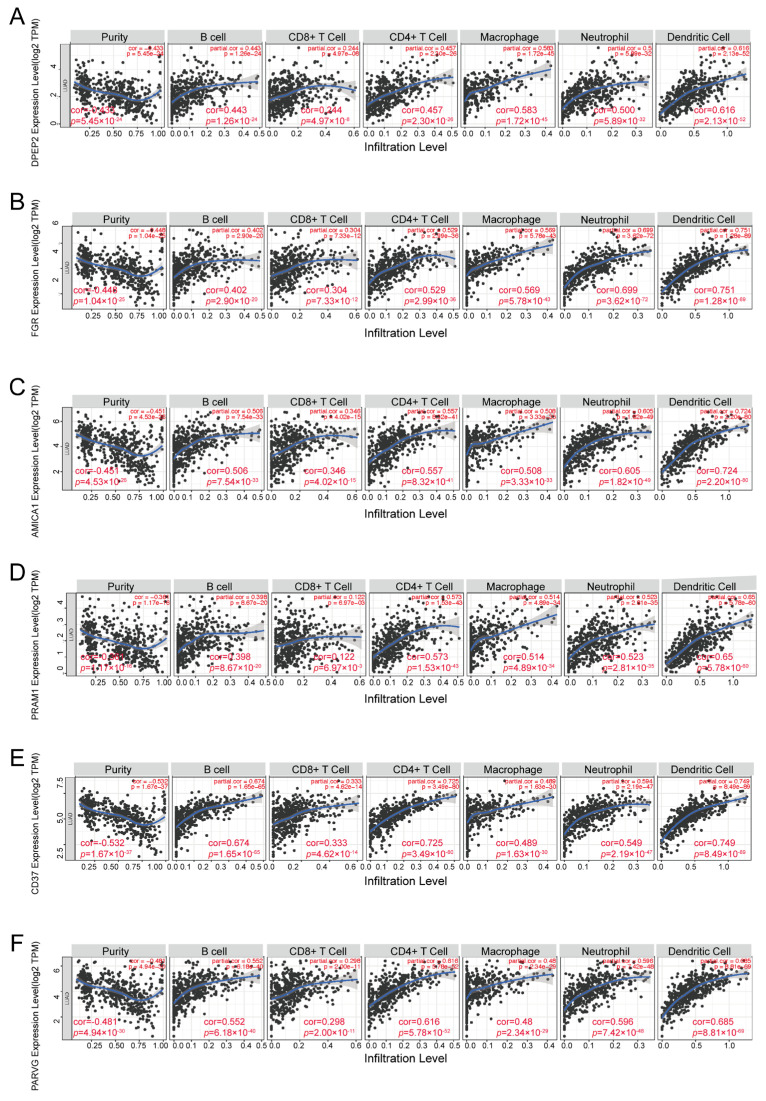
DPEP2 and its co-expressed genes correlate significantly with the level of immune infiltration in LUAD. Correlation of DPEP2 (**A**)**,** FGR (**B**), AMICA1 (**C**), PRAM1 (**D**), CD37 (**E**), and PARVG (**F**).

Using the TIMER database, we investigated the correlation between DPEP2 expression and six types of immune-infiltrating cells in LUAD. Our analysis of the results found that DPEP2 correlated with all six immune cell types, particularly with macrophages and dendritic cells (Figure 4A). Additionally, we explored the correlation between genes significantly associated with DPEP2 and infiltrating immune cells, which revealed that FGR, AMICA1, PRAM1, CD37, and PARVG were co-expressed with DPEP2 and were significantly associated with macrophages and dendritic cells (Figure 4B–F).

**Figure 5:**
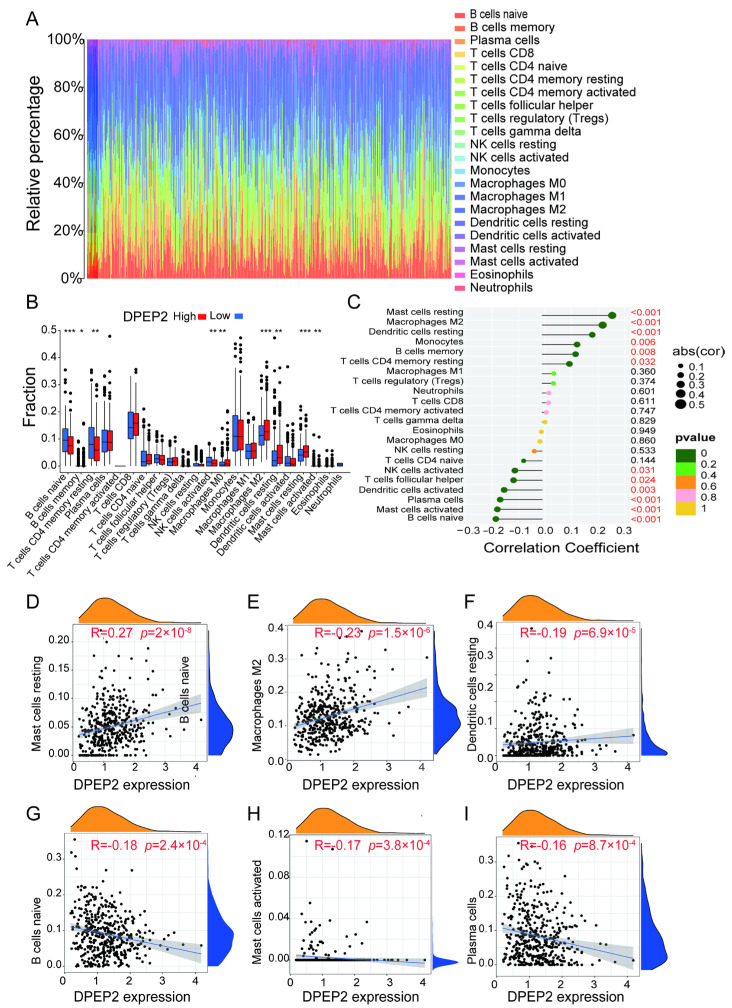
The composition of immune cells assessed by the CIBERSORT algorithm in LUAD and analysis of the correlation between DPEP2 and 22 immune cell types. (**A**) The proportion of immune cell composition in each LUAD sample was determined by the CIBERSORT algorithm. (**B**) Box plots depicting the CIBERSORT scores of 22 immune cells of the DPEP2 high expression group compared to the DPEP2 low expression group. (**C**) Lollipop plot shows the correlation between DPEP2 expression level and CIBERSORT scores of 22 immune cells. (**D**–**F**). Scatter plot demonstrating the correlation of DPEP2 expression with mast cell resting (R = 0.27, *p* = 2 × 10^−8^), macrophage M2 (R = 0.23, *p* = 1.5 × 10^−6^), and dendritic cell resting (R = 0.19, *p* = 6.9 × 10^−5^) (**G**–**I**). Scatter plot demonstrates the correlation of DPEP2 expression with naïve B cells (R = −0.18, *p* = 2.4 × 10^−4^), mast cells activated (R = −0.17, *p* = 3.8 × 10^−4^), and plasma cells (R = −0.16, *p* = 8.7 × 10^−4^) (* *p* < 0.05, ** *p* < 0.01, *** *p* < 0.001).

Using the CIBERSOTE algorithm, we calculated the proportion and abundance of 22 immune cell species in all TCGA-LUAD patients (Figure 5A). Next, LUAD samples were divided into high and low DPEP2 expression groups based on DPEP2 expression levels, with the goal of determining whether the different DPEP2 expression groups differed in terms of the abundance of 22 immune cells in the immune microenvironment of an LUAD tumor. Resting mast cells, M2 macrophages, and resting dendritic cells were increased in the high DPEP2 expression group, while naive B cells, activated mast cells, plasma cells, activated dendritic cells, follicular helper T cells, and activated NK cells were decreased (all *p* values < 0.05) (Figure 5B,C). The scatter plot depicts the relationships between and statistical significance of DPEP2 expression and resting mast cells, M2 macrophages, resting dendritic cells, naive B cells, activated mast cells, and plasma cells (Figure 5D–I). These findings confirm that the expression of DPEP2 in LUAD is closely related to immune cell infiltration.

The ESTIMATH algorithm and the Wilcoxon test were used to examine the significant differences in TME scores (immune score, stromal score, and ESTIMATE score) between the high and low DPEP2 expression groups, with the high DPEP2 expression group having a higher immune score, stromal score, and ESTIMATE score (all *p* values < 0.05) (*p < 0.05, ** *p*< 0.01, *** *p*< 0.001) (Figure S2).

**Figure 6:**
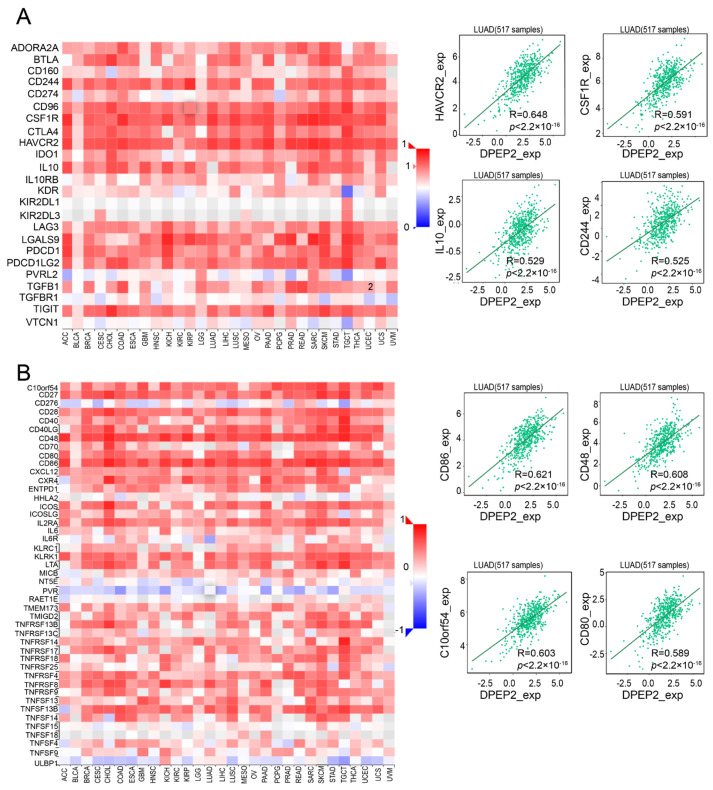
DPEP2 is associated with a variety of immune inhibitors and immune stimulators in LUAD. (**A**) Using the TISIDB database, the heatmap and scatter plots demonstrate the correlation between DPEP2 and immune inhibitors. (**B**) Using the TISIDB database, heatmap and scatter plots demonstrate the correlation between DPEP2 and immune stimulators.

To better understand the relationship between DPEP2 and immune infiltration, we investigated the relationship between DPEP2 expression and various immune inhibitors and immune stimulators in LUAD using the TISIDB database. DPEP2 plays an important role in various immune inhibitors and stimulators, such as HAVCR2, CSF1R, IL10, and CD244 (immune inhibitors, Figure 6A), as well as CD86, CD48, C10orf54, and CD80 (immune stimulators, Figure 6B). Moreover, we found DPEP2 to be associated with multiple immune checkpoints, including LAIR1, HAVCR2, and CD86 (Figure S3). As a result, DPEP2 regulates various immune modulators in LUAD and significantly affects immune infiltration in the LUAD microenvironment.

### 2.6. DPEP2 Is Expressed in Macrophages in the Single-Cell Transcriptome

**Figure 7:**
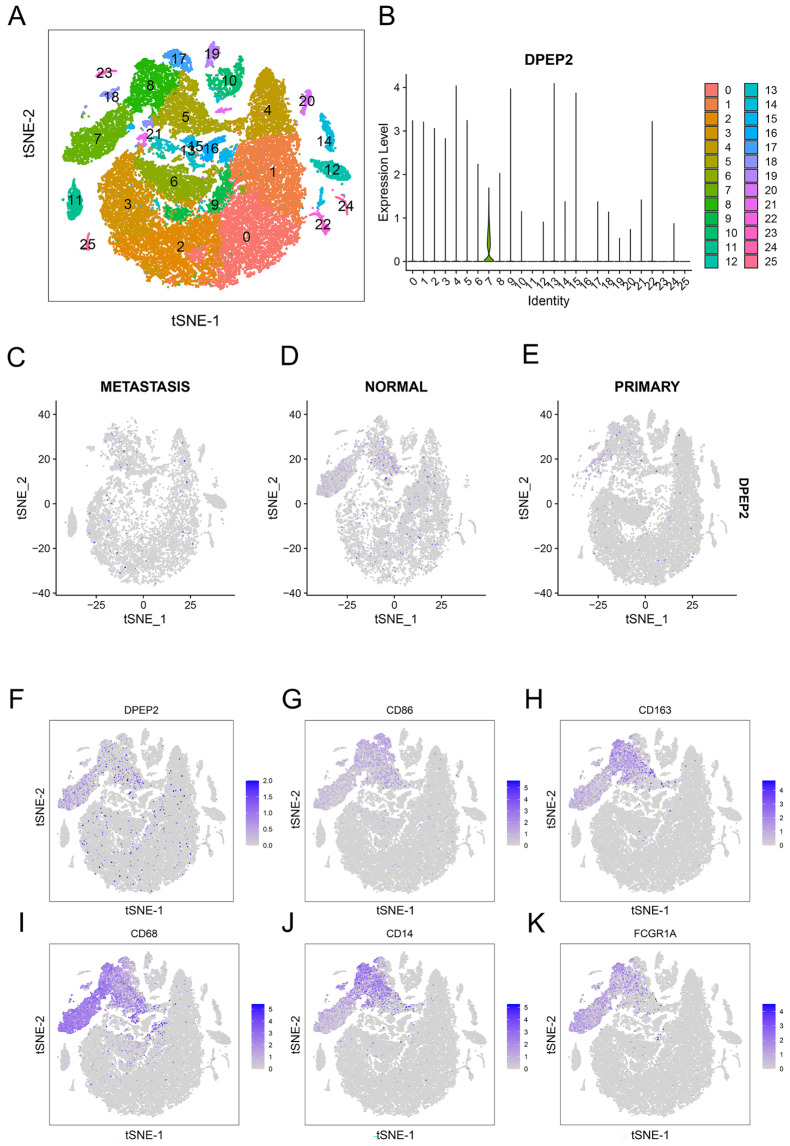
Identification of a DPEP2-enriched cluster and macrophage association in LUAD single-cell analysis. (**A**,**B**) Reduced-dimensional clustering analysis of the single-cell dataset GSE123902 demonstrated that DPEP2 was expressed in the highest abundance in cluster 7. (**C**–**E**) Validation of DPEP2 expression in metastases and normal and primary tumors. (**F**–**K**) Analysis of single-cell GSE123902 data found significant associations between DPEP2 and macrophage-associated marker genes such as CD86, CD163, CD68, CD14, and FCGR1A.

**Figure 8:**
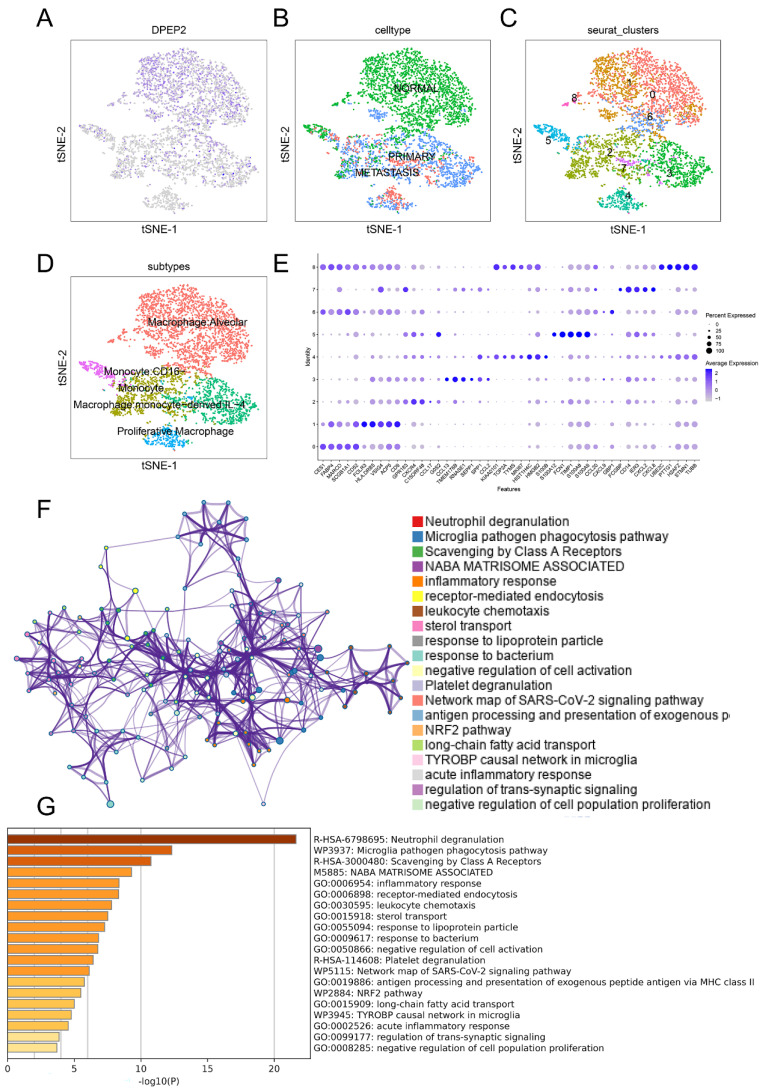
DPEP2 was predominantly enriched in normal alveolar macrophages and was significantly associated with multiple immune-related pathways by analysis of the single-cell dataset GSE123902. (**A**–**C**) Examination of the distribution of DPEP2 in metastatic, primitive malignant, and normal tissues and the associated macrophage clustering. (**D**,**E**) DPEP2 was predominantly enriched in normal tissue-derived alveolar macrophages. (**F**,**G**) Single-cell dataset enrichment analysis of cellular pathways. The top 50 genes differentially expressed in group 7 were involved in immune-related signaling pathways, such as neutrophil degranulation, scavenging by class A receptors, inflammatory response, leukocyte chemotaxis, and receptor-mediated endocytosis.

We analyzed the expression profile of DPEP2 in a single-cell RNA sequencing dataset of LUAD (GSE123902). The sample included four cases of normal lung tissue, eight cases of in situ primary lung tumors, and five cases of metastases. After filtering the data, we obtained a matrix of 23,278 genes expressed by 42,847 cells. After carrying out PCA dimensionality reduction, we obtained 25 cell clusters and presented them in the t-SNE plot (Figure 7A). The bar chart suggests that the highest expression of DPEP2 and the highest concentration were found in cluster 7 (Figure 7B). Furthermore, we discovered that DPEP2 was more abundantly expressed in cell clusters of normal tissue origin versus primary tumor tissue or distant metastatic tissue origin (Figure 7C–E). Finally, we found that DPEP2 was co-expressed with many macrophage markers, such as CD68, CD163, CD14, CD86, and FCGR1A (Figure 7F–K). We extracted DPEP2-enriched cluster 7 cells and obtained t-SNE plots (Figure 8A), and further analysis confirmed that DPEP2 was expressed mainly in cells of normal tissue origin (Figure 8B). In addition, we obtained nine cell clusters, and the analysis revealed that DPEP2 was predominantly expressed in normal alveolar cell macrophages (Figure 8C,D). Figure 8E illustrates the different clusters of cell-associated markers. We further validated DPEP2 expression in GSE162669 and found a higher DPEP2 level in normal alveolar macrophages than in tumor-associated macrophages (Figure S4). Enrichment analysis was conducted by Metascape and was consistent with previous GO, KEGG, and GSEA enrichment analyses in terms of factors such as the inflammatory response and leukocyte chemotaxis. (Figure 8F,G).

### 2.7. Relationship between DPEP2 and Sensitivity to Immunotherapy in LUAD

**Figure 9:**
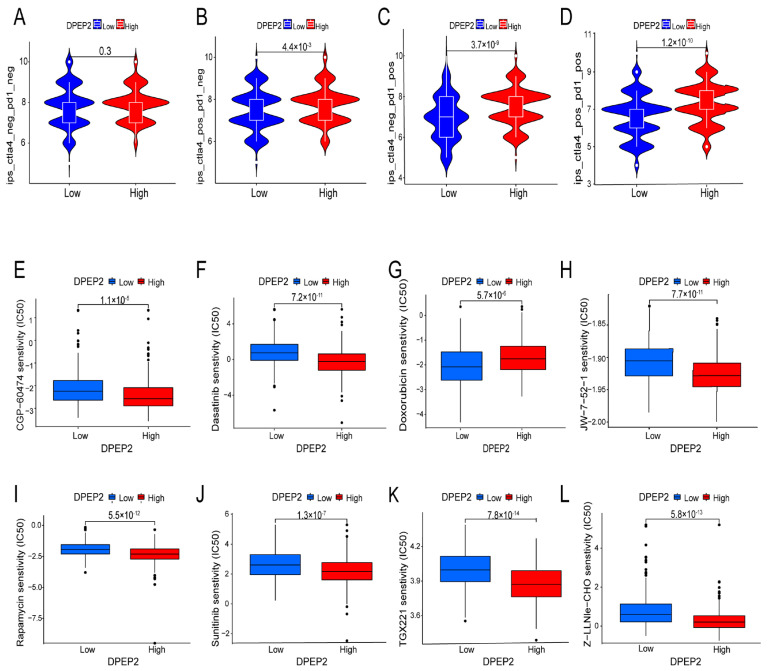
DPEP2 expression significantly correlated with the efficacy of immunotherapy, targeted therapy, and chemotherapy. (**A**–**D**) Sensitivity analysis of immune checkpoint inhibitors in the high and low DPEP2 expression groups. (**E**–**L**) Chemotherapeutic and targeted drug sensitivity analysis in the high and low DPEP2 expression groups.

Our analysis revealed that patients with high DPEP2 expression were more likely to benefit from treatment with PD1 and CTLA4 inhibitors. Specifically, the mean IPS was significantly higher in the high DPEP2 expression group compared to the low DPEP2 expression group in the CTLA4+ PD1−, CTLA4− PD1+, and CTLA4+ PD1+ groups (*p* < 0.05). However, there were no significant differences in the CTLA4− PD1− group (*p* value = 0.3). These findings suggest that patients with high DPEP2 expression may exhibit better responses to PD1 and CTLA4 immunosuppression therapy (Figure 9A–D).

### 2.8. Relationship between DPEP2 and Sensitivity to Drugs in LUAD

We utilized the “pRRophetic” package to predict the IC50 of various chemotherapeutic drugs and targeted therapeutics in both the high and low DPEP2 expression groups. Among the analyzed drugs, sunitinib and dasatinib, both commonly used targeted drugs, as well as CGP-60474, JW-7-52-1, rapamycin, TGX221, and Z-LLNle-CHOD, showed significant differences in IC50 between the high and low DPEP2 expression groups (all *p* < 0.05) (Figure 9E–L).

### 2.9. DPEP2 Is Downregulated in Clinical LUAD Samples

**Figure 10:**
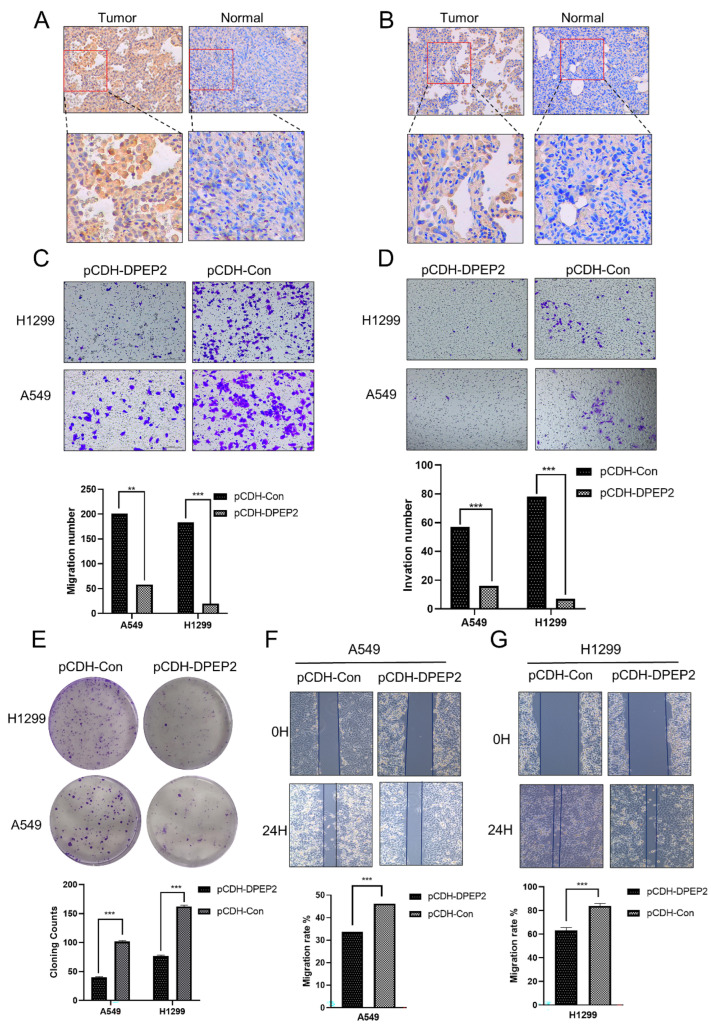
Clinical samples confirmed that DPEP2 expression was downregulated and that overexpression of DPEP2 inhibited the proliferation, invasion, and metastasis of LUAD cells. (**A**,**B**) Immunohistochemistry and clinical samples validated DPEP2 downregulation in LUAD tissues. (**C**,**D**) To study the function of DPEP2 in LUAD, we performed migration and invasion assays in H1299-PCDH, H1299-DPEP2, A549-PCDH, and A549-DPEP2 cells. (**E**) We investigated how DPEP2 affected LUAD cell proliferation in H1299-PCDH, H1299-DPEP2, A549-PCDH, and A549-DPEP2 cells. (**F**,**G**) Using a wound healing experiment, we studied the influence of DPEP2 on the migratory ability of tumor cells in LUAD cell lines. (** *p* < 0.01, *** *p* < 0.001).

More evidence was obtained when we performed IHC staining. We collected 10 matched LUAD tissues and adjacent noncancerous tissues. Compared with those in normal tissues, the protein levels of DPEP2 were lower in LUAD tissues (Figure 10A,B). Furthermore, we exogenously overexpressed full-length human DPEP2 and the control vector PCDH in two LUAD cell lines, A549 and H1299. Exogenously expressed DPEP2 suppressed the migration and invasion of both A549 and H1299 cells, according to the migration and invasion assay results (Figure 10C,D). The subsequent colony formation assay revealed that DPEP2 inhibited proliferation in both cell lines when compared to the empty vector (Figure 10E). In the scratch assay, we confirmed that DPEP2 suppressed the migration of A549 and H1299 cells (Figure 10F,G). Taken together, this evidence suggests that DPEP2 might partially act as a tumor suppressor in LUAD cells.

## 3. Discussion

In this study, we demonstrated that DPEP2 expression was significantly decreased in LUAD tissue compared to adjacent normal tissue. However, the precise functional role and molecular mechanisms of DPEP2 in LUAD remain poorly understood. Previous studies have revealed that DPEP2 acts as an anti-inflammatory factor in delaying the progression of viral myocarditis [12]. Therefore, we propose that DPEP2 might serve similar immunomodulatory and anti-inflammatory functions in LUAD. Further experiments will be conducted to validate our hypothesis.

To gain deeper insights into its role, we analyzed the co-expressed genes of DPEP2 in LUAD using the R package. Our results revealed that DPEP2 was positively correlated with FGR, AMICA1, PRAM1, CD37, and PARVG, with the most significant correlation observed with FGR. Previous research has demonstrated that FGR negatively regulates macrophage phagocytosis, suggesting that DPEP2 may modulate macrophage function in LUAD [15]. Moreover, we found that AMICA1 was also associated with DPEP2 and is a diagnostic and prognostic biomarker that promotes immune cell infiltration in LUAD [16]. Furthermore, GO, KEGG, and GSEA enrichment analyses revealed that genes co-expressed with DPEP2 were significantly enriched in various immune-related pathways, indicating a crucial role of DPEP2 in the immune microenvironment of LUAD. Our findings provide valuable insights into the investigation of the functional mechanisms of DPEP2 in LUAD and offer a theoretical basis for the development of novel therapeutic approaches for LUAD.

The tumor microenvironment plays a crucial role in the development and progression of tumors [17]. Immune cell infiltration is an important component of the tumor microenvironment, and the type and density of infiltrating immune cells can affect tumor prognosis [18]. For example, macrophages are an important class of immune cells in the immune system that can be involved in the immune response by engulfing pathogens, removing cellular waste, and secreting cytokines [19]. In LUAD, macrophages play an important role in the tumor microenvironment [20], but their immune function is often disrupted and suppressed by tumor cells, leading to tumor immune escape and progression [21]. Recently, there has been increasing interest in immunotherapy approaches targeting macrophages for the treatment of LUAD [22]. Such therapeutic strategies include enhancing macrophage function [23]; reversing the M2-type polarization state of macrophages [24]; and targeting immunosuppressive molecules of macrophages, such as PDL1 and CD47 [25]. In this study, we utilized the CIBERSORT algorithm to assess the proportion of 22 immune cell types in LUAD patients. Our analysis revealed a significant correlation between mast cells with high DPEP2 expression and M2 macrophages. Previous studies have suggested that resting mast cells may inhibit tumor development [26], while M2 macrophages are believed to promote tumor development [27]. Therefore, we hypothesize that DPEP2 plays an important role in regulating the function of macrophages and mast cells. Moreover, our analysis of single-cell datasets showed that DPEP2 was significantly enriched in macrophages, especially in normal lung tissues. Furthermore, our hypothesis was further validated by GSE162669 analysis, which showed that the expression of DPEP2 was significantly lower in tumor-associated macrophages compared to alveolar-associated macrophages. These findings provide insight into the potential regulatory mechanisms of DPEP2 in the immune microenvironment of LUAD and may lead to the development of novel therapeutic strategies for LUAD.

Our TISIDB analysis uncovered an intriguing finding: DPEP2 expression in LUAD is positively associated with various immunostimulatory and immunosuppressive factors, including HAVCR2, CSF1R, IL10, CD86, CD48, and CD80. The regulation of immune responses is a complex process [28], and DPEP2 may participate in multiple signaling pathways and regulatory networks that are crucial for immune regulation. It appears that DPEP2 can modulate the immune response in different ways. In some instances, it may upregulate the expression of immunosuppressive factors to maintain immune homeostasis and suppress immune responses. Conversely, it may enhance the immune response by increasing the expression of immunostimulatory factors or by bolstering antitumor or anti-infection immune functions. Furthermore, we postulate that the impact of DPEP2 on the LUAD tumor microenvironment may be influenced by specific cell types or immune environments. In certain cell types or particular immune contexts, DPEP2 expression may positively correlate with the expression of immunosuppressive factors, while in other cell types or diverse immune environments, it may exhibit a positive correlation with the expression of immunostimulatory factors. It is important to acknowledge that these interpretations are based on hypotheses and speculation, underscoring the need for additional research and experimental validation. Future studies will entail more comprehensive experiments and analyses to corroborate and substantiate these hypotheses.

Single-cell sequencing analysis is of paramount importance in tumor research because it offers valuable insights into the cellular heterogeneity and composition of different cell types within tumors [28,29]. Our analysis of single-cell datasets showed that DPEP2 was significantly enriched in macrophages, especially in normal lung tissue. Furthermore, our hypothesis was further validated by GSE162669 analysis, which showed that the expression of DPEP2 was significantly lower in tumor-associated macrophages than in alveolar-associated macrophages. These findings provide insight into the potential regulatory mechanisms of DPEP2 in the immune microenvironment of LUAD and may lead to the development of novel therapeutic strategies for LUAD.

Immunotherapy has become a research hotspot in the treatment of LUAD in recent years [30]. Tumor immunotherapy attacks cancer cells by activating the patient’s own immune system, which has the advantages of strong targeting, stable efficacy, and minimal side effects [31]. Currently, immunotherapy for LUAD mainly includes two types: PD-1/PD-L1 inhibitors and CTLA-4 inhibitors [32]. However, not all patients respond to immunotherapy [33], and it is crucial to identify biomarkers that predict the response to immunotherapy. Several biomarkers have been utilized to develop targeted therapies for LUAD, including EGFR [34], ALK [35], ROS1 [36], BRAF [37], MET [38], and RET [39]. Although progress has been made in targeting these biomarkers, the results are still unsatisfactory. Our study found that patients with high expression of DPEP2 had a higher IPS than those with low expression, suggesting that DPEP2 may be a potential biomarker for predicting the response to immunotherapy in LUAD.

Moreover, monitoring changes in the immune response and DPEP2 expression is critical for assessing the efficacy of various drugs, including chemotherapy and targeted agents such as doxorubicin [40,41], sunitinib [42], and dasatinib [43], which have been tested in clinical trials or are currently used in clinical practice for LUAD treatment. In the future, we will further investigate the potential mechanisms of DPEP2. We expect it to become a predictive biomarker for immunotherapy and a promising therapeutic target for patients with LUAD.

In the last decade, numerous efforts have been made to explore precise and effective treatment targets to halt the progression of LUAD. Considering the critical role of inflammation and immunity in LUAD, more clinical samples and animal models will be required to unravel the specific potential mechanisms in which DPEP2 participates, particularly in regulating immune cells and inflammation in the tumor microenvironment. Additionally, further validation of the differences in drug sensitivity between the high and low DPEP2 expression groups is necessary to gain a better understanding of the pathogenesis of LUAD and provide improved therapeutic options for patients with LUAD.

In this study, we observed frequent and significant downregulation of DPEP2 expression in LUAD in various different datasets, which was further validated by immunohistochemistry analysis showing that DPEP2 was markedly reduced in LUAD and closely correlated with survival and prognosis in LUAD patients. Through the analysis of LUAD single-cell transcriptome datasets, the Tumor Immune Estimation Resource (TIMER), TISIDB, and other databases, we found that DPEP2 plays a pivotal role in shaping the immune microenvironment of LUAD. Interestingly, we also discovered that the expression level of DPEP2 is closely associated with sensitivity to immune checkpoint inhibitors (such as anti-CTLA4 and anti-PD-1), chemotherapeutic agents, and targeted drugs. These findings may contribute to novel and effective therapeutic strategies for the treatment of LUAD.

## 4. Materials and Methods

### 4.1. Data Collection and Integration

To gain a comprehensive understanding of DPEP2 expression in LUAD, we conducted an extensive analysis using the TIMER database. We then used the Genotype-Tissue Expression (GTEx) database to confirm DPEP2 distribution across various normal tissues. The RNA sequencing data from LUAD patients, downloaded from The Cancer Genome Atlas (TCGA) database, were subsequently analyzed using the “limma” package. Additionally, we further investigated DPEP2 expression levels using the Gene Expression Omnibus (GEO) database, UALCAN (http://ualcan.path.uab.edu/index.html, accessed on 30 November 2022), and the Human Protein Atlas (THPA) databases.

### 4.2. Demographic and Clinical Characteristics

X2 statistics were used to examine the relationship between DPEP2 expression and clinical characteristics such as standard TNM staging (clinical and pathological) from the American Joint Committee on Cancer (AJCC) classifications, including factors such as pathologic stage, age, smoking status, and OS events (over survival events).

### 4.3. Survival Analysis, Significant Prognostic Marker Analysis, and Nomogram

We divided TCGA-LUAD samples into two groups of high and low expression based on the median expression value of DPEP2 and then drew survival curves to determine whether the expression of DPEP2 was related to the prognosis of LUAD. Next, the prognostic value of DPEP2 for LUAD was evaluated using the K–M plotter database (https://kmplot.com/analysis/, accessed on 1 December 2022). Univariate and multivariate Cox regression analyses were used to identify independent prognostic factors. Next, to predict the overall survival probability, a nomogram based on univariate and multivariate Cox regression was developed. Calibration plots were then used to evaluate the nomogram’s performance.

### 4.4. Differentially Expressed Gene Analysis

In our study, we grouped TCGA-LUAD patients into high and low DPEP2 expression groups based on the median DPEP2 expression value. To identify differentially expressed genes (DEGs) between these two groups, we utilized the “Limma” R package and set adjusted *p* values of < 0.05 and |log2-fold change (FC)| > 1.5 as the thresholds for DEGs. In addition, to explore the potential co-expression relationships of DPEP2 with other genes, we also performed co-expression analysis.

### 4.5. Functional Enrichment Analysis

The R package “clusterProfiler” was used to perform functional enrichment analyses on the DEGs, including Gene Ontology (GO) and Kyoto Encyclopedia of Genes and Genomes (KEGG) analyses. Gene set enrichment analysis (GSEA) was performed using the R packages “clusterProfiler” and “limma”, and terms with adjusted *p* values < 0.05 and false discovery rates (FDRs) < 0.05 were considered to be statistically significantly enriched function or pathway terms.

### 4.6. Correlation Analysis of DPEP2 and Its Co-Expressed Genes with Immunity in LUAD

The TIMER database was used to assess the relevance of DPEP2 and its related genes to six immune cell types, while the CIBERSORT algorithm was used to investigate the relationship between DPEP2 and 22 different types of human immune cell subpopulations in TCGA-LUAD patients. The ESTIMATE algorithm was used to assess the extent of immune cell and stromal cell infiltration in LUAD tissues. Furthermore, we examined the relationship between DPEP2 expression and immune-related factors, such as various immune inhibitors and immune stimulators, using the TISIDB database. Additionally, we evaluated the correlation between DPEP2 expression and immune checkpoints using the Pearson method.

### 4.7. Single-Cell Transcriptome Analysis

The single-cell RNA sequencing matrix GSE123902 was obtained from 17 donors, including 8 cases of in situ tumor tissue (primary), 4 cases of normal tissue, and 5 cases of metastatic tumor tissue (metastasis). The 10X Genomics platform was utilized for data acquisition. The obtained raw data underwent rigorous quality control and data processing using the “Seurat” R package. First, probe annotation information was obtained and used to map the probes to their corresponding genes. Multiple matches were removed, and for genes with multiple probes, the median values were employed as the representative gene expressions. This resulted in a comprehensive gene expression profile. To ensure the reliability of cell subpopulations, the single-cell data underwent further filtering using the “Seurat” R package. Each gene was required to be expressed in at least 3 cells, and each cell was required to express a minimum of 250 genes. This filtering step aimed to exclude low-quality cells and genes with low expression variability. Mitochondrial gene content and sequencing depth were evaluated to assess cell viability and technical quality. The “PercentageFeatureSet” function was used to calculate the percentage of mitochondrial genes and the number of unique molecular identifiers (UMIs) per cell. Cells with approximately 30% mitochondrial genes and an average of 100 UMIs were retained for subsequent analyses. Data normalization was performed using log-normalization to remove technical biases and to enhance comparability between cells. The “Find Variable Features” function identified highly variable genes based on their dispersion and mean expression, thereby capturing genes that contributed significantly to cell heterogeneity. All genes in the dataset were scaled using the “ScaleData” assay, ensuring that their expression values had similar scales and variances. Principal component analysis (PCA) was then employed for dimensionality reduction, enabling the visualization and exploration of gene expression patterns. Cell clustering was performed using the “FindNeighbors” and “FindClusters” functions, with the resolution parameter set to 0.1. The “FindNeighbors” function calculated cell similarities and constructed a network, while the “FindClusters” function assigned cells into distinct clusters based on their gene expression profiles. Detailed cell annotation was performed by comparing highly expressed genes within each cluster to known gene features and cell marker databases, enabling the identification of cell types within each subgroup.

### 4.8. Expression of DPEP2 and Immunotherapy and Drug Sensitivity Analysis in LUAD

In this study, we aimed to assess the potential predictive value of DPEP2 expression in terms of the efficacy of immune checkpoint inhibitor therapy in LUAD patients. We obtained immunophenoscore (IPS) analyses for both PD1 and CTLA4 inhibitors from the Cancer Immunome (TCIA) database; the higher the IPS value, the more effective the immunotherapy. The Kruskal–Wallis test was used to compare the IPS between the high and low DPEP2 expression groups to predict immunotherapy sensitivity. The half-maximal inhibitory concentration (IC50) of targeted inhibitors was calculated using the R package “pRRophetic”, and the Wilcoxon rank sum test was used to compare the IC50 difference between the high and low DPEP2 expression groups.

### 4.9. Immunohistochemistry

A total of 10 paired LUAD primary tumor and para-cancerous tissues of paraffin-embedded samples were provided by the Pathology Department of Xiangya Hospital of Central South University (Hunan, China). All immunohistochemistry (IHC) procedures were performed with the permission of the Ethical Committee of Xiangya Hospital of Central South University. Tissues were fixed in 10% formalin, paraffin-embedded, cut into 3-mm sections, and mounted on slides. After deparaffinization, rehydration, and microwave antigen retrieval, the slides were incubated at 4 °C overnight with an antibody against DPEP2 (Proteintech, 16466-1-AP, 1:100 working dilution, Rosemont, IL, USA). After that, the slides were incubated with secondary antibody (ImmunoWay, RS0002, with 1:1000 working dilution, Plano, TX, USA) at room temperature for 30 min before being stained with DAB (3,3′-diaminobenzidine) substrate (with 1:20 working dilution) and counterstained with hematoxylin. To validate the positive staining IHC results, five random areas in each tissue sample were microscopically examined and analyzed by at least one experienced pathologist. The average staining scores were subsequently calculated by dividing the positive areas by the total areas. The obtained data were expressed as the mean values ± SDs (standard deviation).

### 4.10. Cell Culture and Transfection Assay for Overexpression

Two human lung cancer cell lines, A549 (CL-0016) and H1299 (CL-0165), were originally purchased from American Type Culture Collection (ATCC) and maintained by the NHC Key Laboratory of Cancer Proteomics of Central South University. All cell lines were used within 3–20 passages after thawing from the primary original stocks, tested every 3 months for mycoplasma contamination, and used only within 3 passages for each experiment. The cells were cultured in DMEM (Dulbecco’s Modified Eagle Medium)/High Glucose medium (Gibco, CT11995500BT, New York, NY, USA) supplemented with 10% FBS (fetal bovine serum) (Biological Industries, 04-001-1C, Kibbutz Beit-Haemek, Israel) and 1% penicillin and streptomycin (Gibco, 15140122). All cells were maintained in 5% CO_2_ at 37 °C with constant humidity. FLAG-tagged human DPEP2 was cloned into the retroviral vector (pCDH; CD513B-1). pCDH-DPEP2 was co-transfected with vectors expressing pMD2 G and pSPAX2 genes by Lipo2000 (Invitrogen, 11668-027, Carlsbad, CA, USA) into HEK293T cells. After retroviral harvest, A549 and H1299 cells were infected with the prepared virus for 48 h and screened using puromycin (Selleck, CL13900; 2 μg/mL final concentration, Houston, TX, USA) for at least 2 weeks. All vectors and stable cell lines were deposited in the NHC Key Laboratory of Cancer Proteomics, Xiangya Hospital.

### 4.11. Wound Healing Assay

Cells were cultured until they reached over 90% confluence in 6-well plates. Subsequently, the cell layers were scratched using a 10 μL sterile pipette tip to generate wound gaps. Then, the cells were washed twice with micron filter-sterilized PBS (phosphate-buffered saline) buffer and cultured in the incubator at a constant temperature and humidity level. The wound gap of the tumor cells was photographed at 0 h and 24 h and then analyzed by measuring the distance of the migrating cells in five different areas of each wound sample.

### 4.12. Transwell Migration and Invasion Assay

For the transwell cell migration assay, a total of 3 × 10^4^ cells were first seeded on the upper transwell chambers with over 300 μL serum-free culture medium (DMEM, Gibco C11995500BT), and the lower chambers were supplied with over 700 μL medium containing 10% FBS (fetal bovine serum, New Zealand, A6904FBS). Following a 24 h incubation period, the tumor cells that had migrated through the membranes (Corning, 3413, New York, USA) were fixed with methanol and stained with 1% crystal violet (Solarbio, G1064, Beijing, China). Photographs of tumor cells that migrated through transwell chambers were taken from five random fields via microscopy (Leica, DM750, Wetzlar, Germany). Quantitative analysis of cell migration was performed by ImageJ (version 1.8.0). For the invasion assay, a total of 1 × 10^5^ cells were seeded in the upper chambers of the transwell plate and analyzed as described above.

### 4.13. Statistical Methods

Using summary statistics, we described the patients’ baseline characteristics and treatment methods. The differences between qualitative and continuous variables were examined using X^2^ statistics, the t test, and analysis of variance (ANOVA). The Kaplan–Meier plot and its associated log-rank test were used to compare the survival rates of the two patient groups (high DPEP2 expression vs. low DPEP2 expression). All tests described above were two-tailed, with *p* values less than 0.05.

## 5. Conclusions

In conclusion, DPEP2 shows great promise as a potential biomarker for predicting the response to immunotherapy in LUAD. The downregulation of DPEP2 in LUAD has been validated through multiple databases, clinical samples, and immunohistochemical results. Moreover, the tumor-suppressive effects of DPEP2 have been further confirmed in LUAD cell lines. Notably, DPEP2 encodes a protein product of a dipeptidase, which plays a role in the final steps of intracellular protein degradation, including LTD4. Further investigation of the interaction patterns and potential substrates of DPEP2 in LUAD is interesting. These findings provide important insights for developing novel therapeutic strategies for LUAD. Overall, our study highlights the potential of DPEP2 as a biomarker for predicting the response to immunotherapy and as a promising target for developing more effective treatments for LUAD.

## Figures and Tables

**Table 1:** Correlation of DPEP2 expression with the clinical characteristics of TCGA-LUAD.

Characteristic	Low Expression of DPEP2	High Expression of DPEP2	*p*
n	267	268	
T stage, n (%)			0.004
T1	72 (13.5%)	103 (19.4%)	
T2	149 (28%)	140 (26.3%)	
T3	31 (5.8%)	18 (3.4%)	
T4	14 (2.6%)	5 (0.9%)	
N stage, n (%)			0.038
N0	162 (31.2%)	186 (35.8%)	
N1	50 (9.6%)	45 (8.7%)	
N2	47 (9.1%)	27 (5.2%)	
N3	1 (0.2%)	1 (0.2%)	
M stage, n (%)			0.557
M0	187 (48.4%)	174 (45.1%)	
M1	15 (3.9%)	10 (2.6%)	
Pathologic stage, n (%)			0.011
Stage I	129 (24.5%)	165 (31.3%)	
Stage II	65 (12.3%)	58 (11%)	
Stage III	53 (10.1%)	31 (5.9%)	
Stage IV	15 (2.8%)	11 (2.1%)	
Age, n (%)			<0.001
<=65	147 (28.5%)	108 (20.9%)	
>65	110 (21.3%)	151 (29.3%)	
Smoker, n (%)			0.003
No	25 (4.8%)	50 (9.6%)	
Yes	236 (45.3%)	210 (40.3%)	
OS event, n (%)			0.008
Alive	156 (29.2%)	187 (35%)	
Dead	111 (20.7%)	81 (15.1%)	

## Data Availability

All data and R scripts in this study are available from the corresponding author upon reasonable request. All authors have read and approved the final manuscript. Publicly available datasets were analyzed in this study, which can be found in The Cancer Genome Atlas (https://portal.gdc.cancer.gov/, accessed on 27 May 2023), Genotype-Tissue Expression (https://www.gtexportal.org/home/, accessed on 27 May 2023), and Gene Expression Omnibus (GSE10072, GSE43458, GSE44077, GSE116959, GSE123902).

## Supplementary Materials

The following supporting information can be downloaded at https://www.mdpi.com/article/10.3390/ph16060871/s1, Figure S1: Calibration curve of the nomogram in the TCGA-LUAD cohort, Figure S2: Stroma, immune, and ESTIMATE scores in the DPED2-high and DPED2-low groups in the TCGA-LUAD dataset, Figure S3: The correlation between DPEP2 and multiple immune checkpoints is shown in the heatmap, Figure S4: DPEP2 expression is downregulated in tumor-associated macrophages, as shown by the bar graphs.

## Abbreviations

LUAD	Lung adenocarcinoma
NSCLC	Non-small cell lung cancer
LTD4	Leukotriene D4
DPEP2	Dipeptidase 2
LTE4	Leukotriene E4
TIMER	The Tumor Immune Estimation Resource
GTEx	Genotype-Tissue Expression
TCGA	The Cancer Genome Atlas
GEO	Gene Expression Omnibus
THPA	The Human Protein Atlas
AJCC	American Joint Committee on Cancer
OS event	Over survival event
DEGs	Differentially expressed genes
GO	Gene Ontology
KEGG	Kyoto Encyclopedia of Genes and Genomes
GSEA	Gene Set Enrichment Analysis
UMIs	Unique molecular identifiers
PCA	Principal component analysis
IPS	Immunophenoscores
TCIA	The Cancer Immunome Database
IC50	The half-maximal inhibitory concentration
IHC	Immunohistochemistry
ATCC	American Type Culture Collection
DMEM	Dulbecco’s Modified Eagle Medium
FBS	Fetal bovine serum
PBS	Phosphate buffered saline
ANOVA	Analysis of variance
