# Supplementary material for: Integrated Single-Cell and Transcriptome Sequencing Analyses Identify Dipeptidase 2 as an Immune-Associated Prognostic Biomarker for Lung Adenocarcinoma
Source: Pharmaceuticals (Basel). 2023 Jun 12;16(6):871. doi: 10.3390/ph16060871 (PMC10302781; doi:10.3390/ph16060871)
Supplement: Supplementary file 1 [file pharmaceuticals-16-00871-s001.zip › pharmaceuticals-2386988-supplementary.pdf]

Supplementary materials

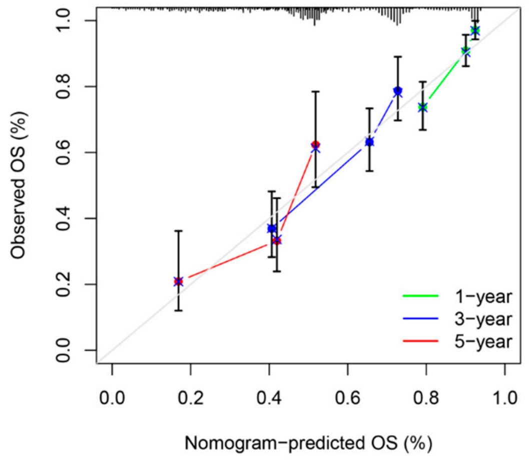

Figure S1. Calibration curve of the nomogram in the TCGA-LUAD cohort. The predicted survival probability produced by the nomogram is the x-axis, and the actual survival is the y-axis. Close alignment with the 45-degree diagonal represents good estimation.

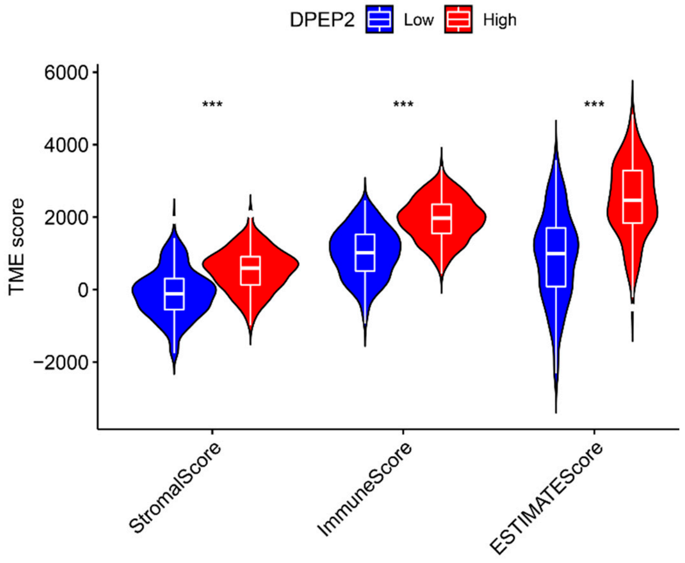

Figure S2. Stroma, immune, and ESTIMATE scores in the DPED2-high and DPED2-low groups in the TCGA-LUAD dataset. The DPEP2 high expression group had higher stroma, immune, and ESTIMATE scores.

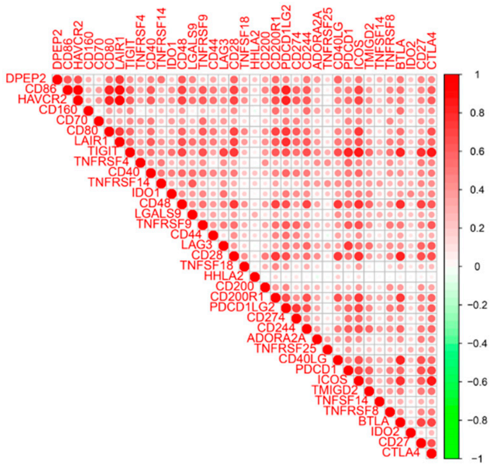

Figure S3. The correlation between DPEP2 and multiple immune checkpoints is shown in the heatmap.

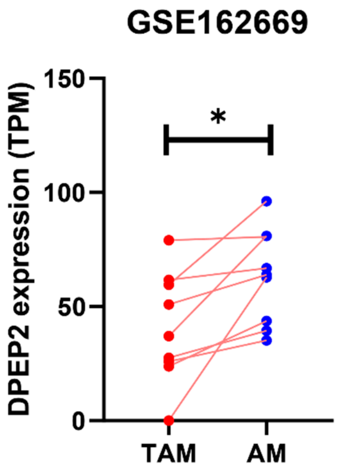

Figure S4. DPEP2 expression is downregulated in tumor-associated macrophages, as shown by the bar graphs (TAM: tumor-associated macrophages, AM: alveolar macrophages).
